# Full-length transcriptome characterization of *Platycladus orientalis* based on the PacBio platform

**DOI:** 10.3389/fgene.2024.1345039

**Published:** 2024-01-18

**Authors:** Ting Liao, Linyi Zhang, Ye Wang, Liqin Guo, Jun Cao, Guobin Liu

**Affiliations:** Institute of Forestry and Pomology, Beijing Academy of Agriculture and Forestry Sciences, Beijing, China

**Keywords:** *Platycladus orientalis*, full-length transcriptome, SMRT sequencing, functional annotation, plant hormone

## Abstract

As a unique and native conifer in China, *Platycladus orientalis* is widely used in soil erosion control, garden landscapes, timber, and traditional Chinese medicine. However, due to the lack of reference genome and transcriptome, it is limited to the further molecular mechanism research and gene function mining. To develop a full-length reference transcriptome, tissues from five different parts of *P. orientalis* and four cone developmental stages were sequenced and analyzed by single-molecule real-time (SMRT) sequencing through the PacBio platform in this study. Overall, 37,111 isoforms were detected by PacBio with an N50 length of 2,317 nt, an average length of 1,999 bp, and the GC content of 41.81%. Meanwhile, 36,120 coding sequences, 5,645 simple sequence repeats (SSRs), 1,201 non-coding RNAs (lncRNAs), and 182 alternative splicing (AS) events with five types were identified using the results obtained from the PacBio transcript isoforms. Furthermore, 1,659 transcription factors (TFs) were detected and belonged to 51 TF families. A total of 35,689 transcripts (96.17%) were annotated through the NCBI nr, KOG, Swiss-Prot and KEGG databases, and 385 transcript isoforms related to 8 types of hormones were identified incorporated into plant hormone signal transduction pathways. The assembly and revelation of the full-length transcriptome of *P. orientalis* offer a pioneering insight for future investigations into gene function and genetic breeding within *Platycladus* species.

## 1 Introduction

As one of the most important coniferous species in China, *P*. *orientalis* (L.) Franco belongs to the Cupressaceae family and it is widely used for sand fixation, wind protection, preventing of soil erosion, and afforestation in the barren mountains of northern China. It’s highly resilient to extreme environments, and can withstand extreme temperatures from −35°C to 45°C. Moreover, because it is evergreen and resistant to pruning, *P. orientalis* is commonly used as a hedge and street tree in landscaping. The branches, leaves and seeds of *P. orientalis* can be used as medicine, and it contains volatile oils, fatty acids, vitamins, ketones and other substances. Essential oils and spices for disinfection can be extracted from the needles and trunks of *P. orientalis* (Guleria et al., 2008; Wang et al., 2012).

The DNA content in the genome of *P. orientalis* was approximately 10.46 pg, corresponding to the genome size of approximately 10.23 Gb (Hu et al., 2016). The number of chromosomes in *P. orientalis* was *n* = 11, and all were equibrachial, except 1 to 2 chromosomes with unequal arms (Sax and Sax, 1933). Karyotype analysis of *P. orientalis* showed that the total length of the chromosomes was 97.71 µm. They had a middle centromere and belong to the symmetrical karyotype. One pair of chromosomes had a stable secondary constriction (Li and Xu, 1984). The karyotype analysis of 10 genera (22 species) of Thujoideae indicated that the five Southern Hemisphere genera (*Callitris*, *Actinostrobus*, *Libocedrus*, *Microbiota*, and *Widdringtonia*), as well as *Platycladus* and *Tetraclinis,* were the most primitive, while *Thujopsis* and *Thuja* were the most evolved, with *Calocedrus* in the middle, according to Li et al. (1996). The genome of coniferous plants was characterized by evolutionary decline in the number of coding genes and accumulation of DNA in non-coding regions, which was far from the evolution of angiosperms (Ritland, 2012). Therefore, the study of coniferous plants is very important and significant for genetic evolution. Owing to the lacing of genome information of *P. orientalis*, to date, the utilization of *P. orientalis* breeding resources is still at the primary stage. Research on *P. orientalis* mainly focuses on seedling breeding, propagation and field cultivation techniques, ecological function, and disease and pest control (Gadek and Quinn, 1985; Gadek and Quinn, 1988; Sugihara, 1992; Kumrann, 1994). Our research group has performed some cytological studies on floral formation, development and rooting mechanisms of *P. orientalis* (Liao et al., 2021; Liu et al., 2021), but there are little studies focus on molecular biology.

Due to the huge size of genome and complex genetic backgrounds in *P. orientalis*, it is difficult and costly to assemble and analyze the whole genome sequencing (WGS). Up to now, RNA sequencing based on high throughput platforms can also obtain high-quality reference transcriptome sequences. This technique is widely used in non-model species lacking reference genomes, and it is critical for understanding the genetic relationships between genotypes and phenotypes in these species (Conesa et al., 2016). In recent years, the third-generation high-throughput sequencing technology has been successfully applied on both animals and plants with functional genome research based on SMRT sequencing. Third-generation sequencing is an important way to develop genomic resources and molecular markers, and this technology has led to the establishment of transcriptome databases for a variety of plants without reference genomes. Owing to the limitation of the transcriptome read length (PE150) based on second-generation sequencing platforms, the obtained sequencing fragments need to be spliced; there are more chimeras in the process of transcript assembly, and complete transcript information cannot be accurately obtained, which greatly reduces the accuracy of analysis, such as expression levels, variable splicing, and gene fusion. Compared with the traditional second generation sequencing technology, the third-generation sequencing technology can produce large data throughput, long sequence read length (average 15 kb), and longer transcript length, which less require sequence splicing and assembly, and prevents more splicing errors, so that higher quality transcript sequences can be obtained. This is more improvement of mRNA sequence structure and gene expression research, so as to greatly improving the integrity of gene sequence splicing and the accuracy of functional annotation (Sharon et al., 2013; Rhoads and Au, 2015; Stadermann et al., 2015; Wang et al., 2019a; Chao et al., 2019). Third-generation sequencing technology also benefits to the discovery of new genes and analysis of homologous, molecular markers, gene families, and allelomorphic genes (Wang et al., 2019b; Byrne et al., 2019) and has been utilized for some woody species, including *Cinnamomum porrectum* (Qiu et al., 2019), *Torreya grandis* (Lou et al., 2019), *Olea europaea* (Rao et al., 2019), *Vitis vinifera* (Minio et al., 2019), *Madhuca pasquieri* (Dubard) Lam (Kan et al., 2020), *Rhododendron lapponicum* (Jia et al., 2020), *Cephalotaxus oliveri* (He et al., 2021) and *Chosenia arbutifolia* (He et al., 2022).

Despite its important function in landscaping and ecological restoration, thus far, no studies had been reported about the full-length transcriptome characterization in *P. orientalis*. In this research, the full-length transcriptome of *P. orientalis* was sequenced by SMRT sequencing. To investigate the cone development mechanism of *P. orientalis* and ensure wide transcript coverage, five different tissues including root, stem, needle leaf, seeds and cones from four different developmental periods were mixed for transcriptome analysis. Therefore, full-length sequence prediction, SSR, TF, lncRNA, and AS event prediction and gene function were predicted and analyzed through the procured full-length transcriptome data. Furthermore, transcript isoforms involved in plant hormone signal transduction were analyzed to determine the types and expression of hormone-related genes in different cone developmental stages of *P. orientalis*. The results of this work provide insight on molecular marker development, genetic function, new genes detecting, genetic classification and evolution, which can promote a deeper genetic breeding of *P. orientalis*.

## 2 Materials and methods

### 2.1 Plant materials

Plant materials of *P. orientalis* used in this research were grown in the coniferous plant resource nursery at the Institute of Forestry and Pomology, Beijing Academy of Agriculture and Forestry Sciences, Beijing, China. The excellent varieties of trees named “dieye” that selected by our research group were used as materials. The tree was approximately 15 m high and 25 years old. According to the meteorological record, the climate of the nursery was typical of subhumid continental monsoon climate, and the soil was alkaline soil (pH 7.1–8.2). The average annual temperature was 11°C∼13°C, and the average annual rainfall was about 626 mm (Liao et al., 2021). From June to September, the roots, stems, needles, cones and seeds of *P. orientalis* were sampled. In order to ensure the integrity of sequencing and avoid the specificity of gene expression in samples at different developmental stages, the healthy roots, stems and leaves were taken from the most vigorous growth period in July, and the seeds were taken from the September until mature. According to the cones formation and development period determined by paraffin section anatomy, male and female cones were respectively taken from four different developmental stages in *P. orientalis*, including the pre-sex differentiation, differentiation, post-differentiation and dormancy periods according to Liao et al. (2021). The samples were quickly frozen in liquid nitrogen and were brought back to the lab stored at −80°C for subsequent experiments.

### 2.2 RNA extraction, library construction and sequencing

Total RNA of all samples was extracted by TRIzol reagent (Invitrogen, Carlsbad, CA, United States) for subsequent analysis following the procedure provided by the kit. The purity (OD260/280 ratio) and integrity of total RNA were detected by Nanodrop (Thermo Fisher) and Agilent2100 bioanalyzer (Agilent Technologies, Palo Alto, CA, United States). At the same time, agarose gel electrophoresis was used to analyze the degree of RNA degradation and contamination. After RNA detection, the mRNA containing poly(A) enriched by Oligo(dT) was reverse transcribed into cDNA by SMARTer PCR cDNA Synthesis Kit (TaKaRa Bio, Inc., Kusatsu, Shiga, Japan), and then the SMRT bell library was constructed by large-scale PCR amplification initially. Subsequently, the full-length cDNA was subjected to terminal repair and exonuclease digestion. The results were re-screened by BluePippin, and the sequencing library was finally obtained.

### 2.3 Preprocessing of SMRT sequencing data

The qualified library was sequenced using the Pacbio Sequel platform, and the original data were processed using the official PacBio software SMRTlink with default parameters to obtain Subreads sequences. The high-quality circular consensus sequences (CCSs) were extracted from subread BAM files. According to whether the sequence contained 5′ primers, 3′ primers and poly(A) structures, the sequences were divided into full-length sequence (FL reads) and non-full-length sequence. Afterward, consensus sequence (unpolished consensus isoforms) was obtained by clustering full-length non-chimeric (FLNC) sequences of the same transcript using the same type clustering (ICE) algorithm. Then, the polished consensus sequences were obtained for subsequent analysis. CD-HIT v 4.7 with default parameters was used to eliminate the redundancy of the consistent sequences, and the sequences whose similarity were more than 99% were combined. The method of local alignment was adopted, in which, for short sequences, the alignment rate must reach 99%, and the number of unmatched bases should be less than 30 bp. For longer sequences, the alignment ratio must be 90%, and the number of bases that did not match must be less than 100 bp. Finally, the full-length transcriptome of the sample was obtained.

### 2.4 Prediction of CDSs, SSRs, TFs, lncRNAs and AS events

The obtained sequences were predicted and analyzed with ANGEL software for coding sequence (CDS) (Shimizu et al., 2006). MISA software (http://pgrc.ipk-gatersleben.de/misa/) was used for SSR sequence detection, and the number of repetitions of single nucleotides was 10, dinucleotide was 6 (Beier et al., 2017). The minimum repeats number of trinucleotide, tetranucleotide, pentanucleotide and hexanucleotide was 5. Transcription factor (TF) prediction was performed using iTAK software and.Plant TFdb (http://planttfdb.cbi.pku.edu.cn/) (Zhang et al., 2015) to explore TF families in *P. orientalis*. Coding and non-coding transcripts were categorized using the CNCI (Sun et al., 2013) and CPC (Kong et al., 2007) software programs. And AS events analysis was performed using SUPPA (Gael et al., 2015) tool in transcript isoforms of *P. orientalis*.

### 2.5 Functional annotation

For the obtained high-quality sequences, BLAST against was performed to evaluate sequence similarities with other species with an E-value threshold of 10^−5^ through the NCBI non-redundant protein (nr) database (http://www.ncbi.nlm.nih.gov), NCBI non-redundant nucleotide sequence (Nt, ncbi-blast-2.7.1+) database, COG/KOG database (http://www.ncbi.nlm.nih.gov/COG), Kyoto Encyclopedia of Genes and Genomes (KEGG) database (http://www.genome.jp/kegg), and Swiss-Prot protein database (http://www.ExPASy.ch/sprot) by using the BLASTx v2.2.31 program (http://www.ncbi.nlm.nih.gov/BLAST/). Then, the cluster of orthologous groups of proteins, gene ontology (GO) and KEGG were used for homologous gene function prediction, classification and metabolic pathway analysis.

## 3 Results

### 3.1 General properties of PacBio SMRT sequencing

**FIGURE 1:**
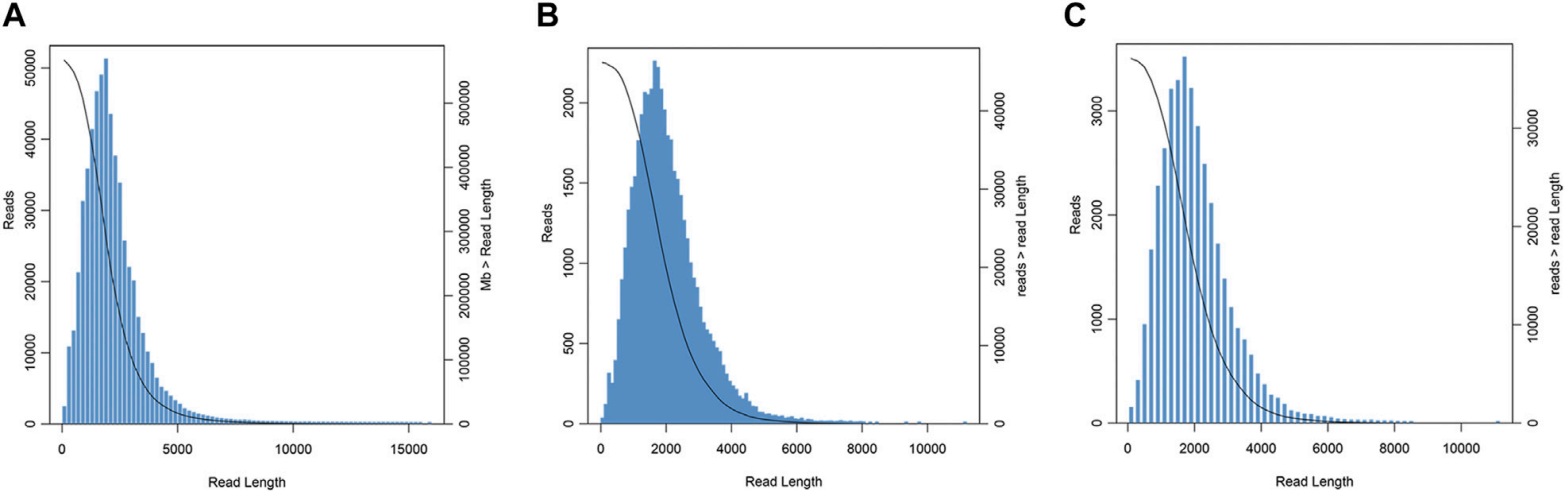
Length distribution of circular consensus sequences (CCSs) **(A)**, consensus isoforms **(B)**, and isoform sequences **(C)** obtained from PacBio single-molecule long-read (SMRT) sequencing in *P. orentalis*.

A total of 43,028,157,120 bp data and 26,770,800 subreads, with a mean length of 1,607 bp and an N50 of 2,106 bp, were obtained by using the PacBio Sequel sequencing platform. CCS was a sequence with low error rate obtained by the correction of multiple sequencing results. A total of 566,812 CCSs were obtained with a mean length of 2,149 bp and an average depth of 41 passes as showed in Figure 1A. Furthermore, 45,805 high-quality sequences and 389 low-quality sequences were obtained by the ICE and Quiver algorithms. The length distribution of the consensus isoforms is shown in Figure 1B. After the clustering and correction of reads, removing redundancy from the consistent sequence was performed. And finally, the full-length transcriptome of *P. orientalis* was obtained. There were 37,111 isoforms with the total length of 74,191,193 bp, the average length of 1,999 bp, the N50 length of 2,317 bp, and the GC content of 41.81% (Figure 1C).

### 3.2 Prediction of CDSs, SSRs, TFs, lncRNAs and AS events

**FIGURE 2:**
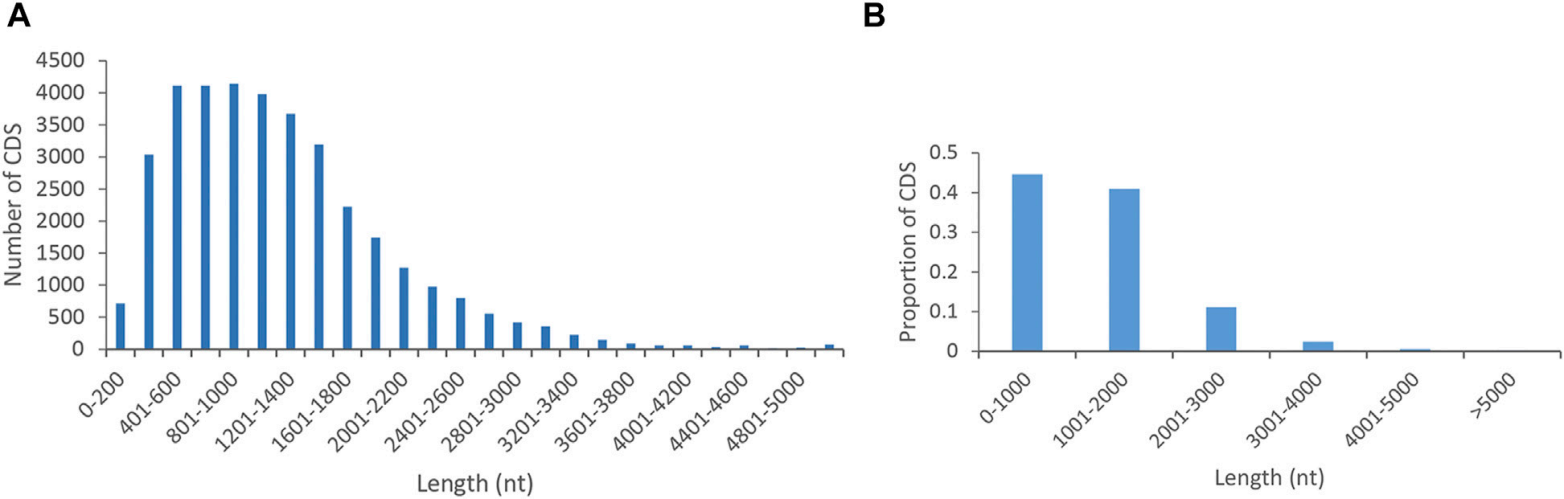
Distribution of CDS lengths **(A)** and proportions **(B)** in *P. orentalis*.

The numbers and proportions of the length distribution of proteins encoded by CDS regions in *P. orientalis* were shown in Figure 2. A total of 36,120 coding sequences were predicted by PacBio Sequel. CDS lengths ≤2,000 bp accounted for 85.6% (30,925), followed by those from 2,000 to 3,000 bp (4,025; 11.1%) and >3,000 bp (1,170; 3.2%).

**FIGURE 3:**
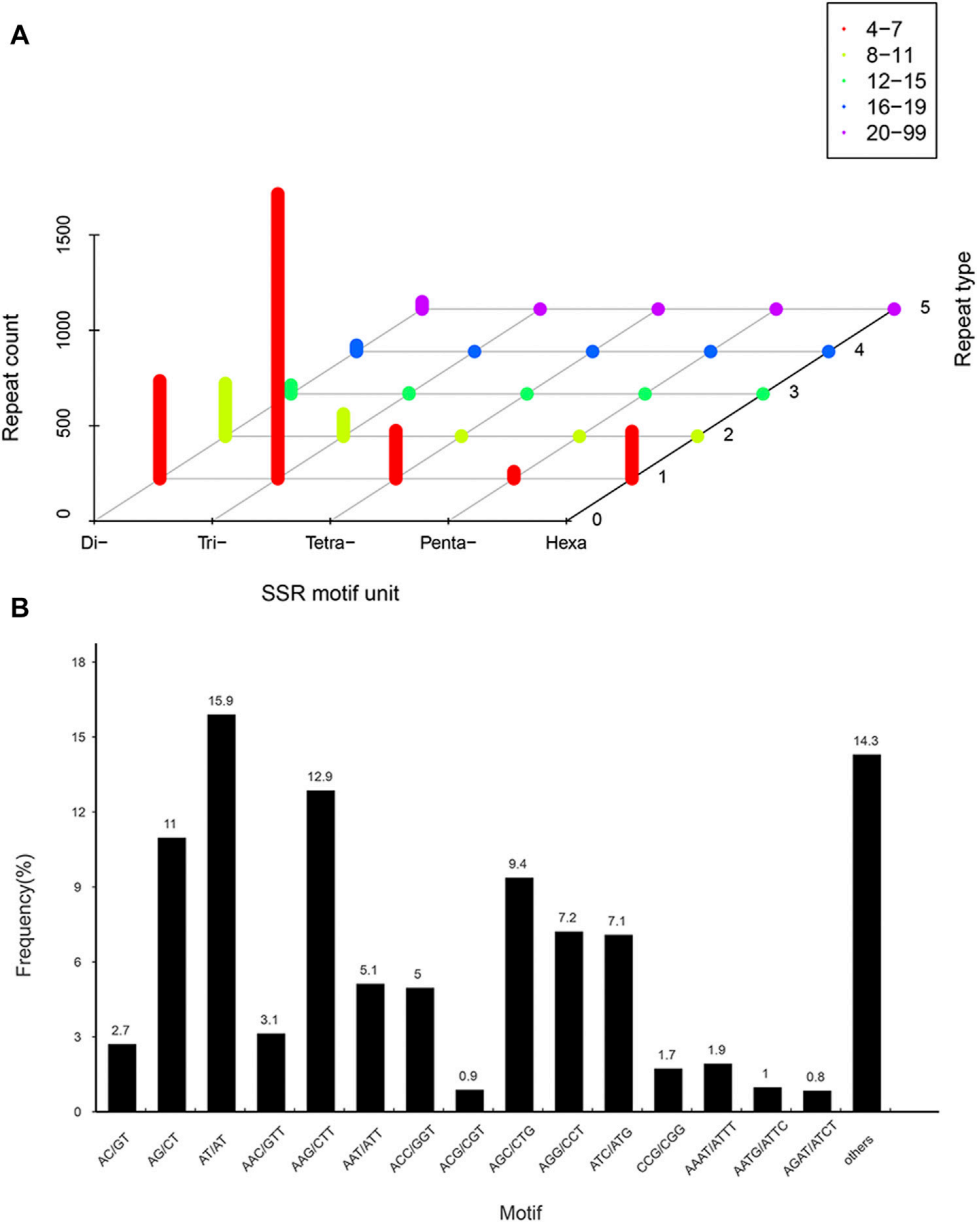
Distribution of simple sequence repeat (SSR) nucleotide classfication in *P. orientalis* transcriptom. **(A)** Distribution statistics of six types of SSRs in *P. orientalis*. **(B)** Proportions of SSRs with different types among tandem repeat elements in total SSRs.

SSRs in the transcriptome were detected by MISA 1.0. A total of 3,063 identified SSRs and 2,582 SSR-containing sequences were detected in 37,111 isoforms. Of these, 347 transcripts contained more than one SSR, and 236 contained compound SSRs. Trinucleotide repeat motifs (1,615) with 4–15 repeats were the most abundant, accounting for 52.7%. Then followed by dinucleotide (908; 29.6%), with 4–99 repeats, tetranucleotide (254; 8.3%), with 4–11 repeats, and hexanucleotide (249; 8.1%) repeat motifs, with 4–11 repeats. However, pentanucleotide repeat motifs (37; 1.2%) with 4–7 repeats were the least abundant (Figure 3A). Furthermore, among the dinucleotide repeats, AT/AT (487, 15.9%) was the most frequent, followed by AG/CT (336, 11%). Among the trinucleotide repeats, AAG/CTT (394, 12.9%) was the most abundant, followed by AGC/CTG (287, 9.4%), AGG/CCT (221, 7.2%) and ATC/ATG (217, 7.1%) (Figure 3B). Among the tetra-, penta- and hexanucleotide repeats, AAAT/ATTT, TAGTT/TATTT, and TGCAGC/CGCCTC were the most abundant, with the repeat numbers of 59, 10 and 12, respectively.

**FIGURE 4:**
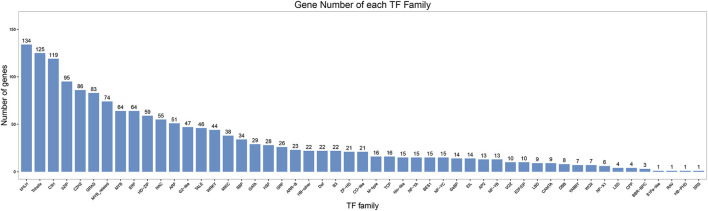
Statistics of transcription factor (TF) families predicted by PacBio in *P. orientalis.*

As nodal regulatory genes, TFs played important roles in plant growth, flowering and development. The assemble and predicted protein sequences were compared with the TF databases using the iTAK software. In total, 1,659 TFs were identified and could be classified into 51 TF families. Of which, the top 10 TF families belonged to bHLH (134, 8.1%), trihelix (125, 7.5%), C3H (119, 7.2%), bZIP (95, 5.7%), C2H2 (86, 5.2%), GRAS (83, 5.0%), MYB related (74, 4.4%), MYB (64, 3.9%), ERF (64, 3.9%), and HD-ZIP (59, 3.6%) (Figure 4).

**FIGURE 5:**
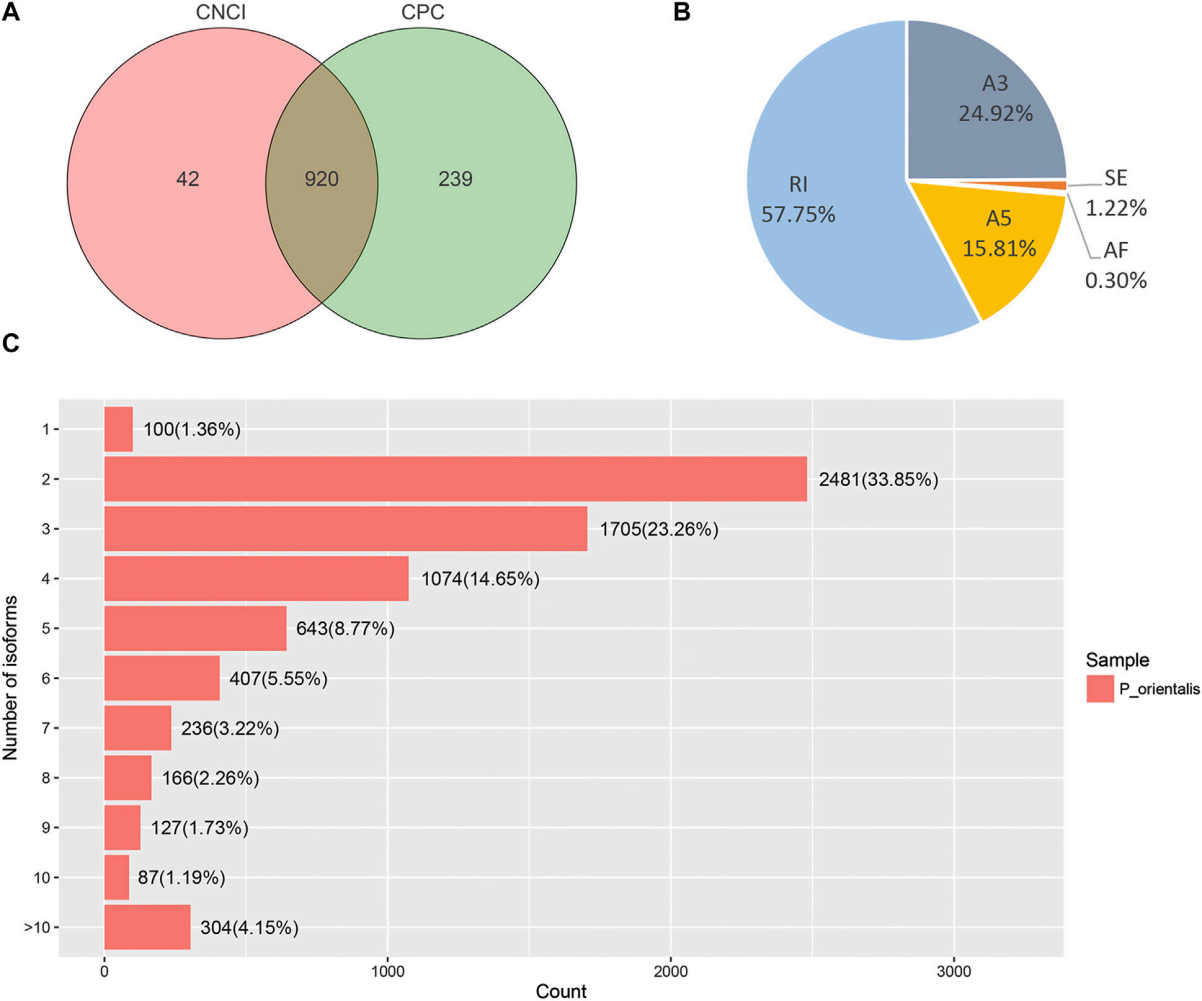
Analysis of the Candidate lncRNAs and AS events in *P. orentalis* transcriptome. **(A)** Candidate lncRNAs identified by CNCI and CPC. **(B)** The number and percentage of AS events. A3, alternative 3′ splice site; A5, alternative 5′ splice site; AF, alternative first exon; RI, retained intron; SE, skipping exon. **(C)** Statistics of the isoforms number.

To predict lncRNAs from putative protein-coding RNAs, CNCI and CPC were used to achieve this purpose from unknown transcripts. The CNCI tool identified 962 lncRNAs and CPC identified 1,159 lncRNAs, respectively. In total, 1,201 total lncRNAs and 920 co-lncRNAs were predicted by the two methods (Figure 5A).

A total of 182 alternative splicing (AS) events of five types were identified using the results obtained for PacBio transcript isoforms. Among them, RI at 190 was the main AS event, accounting for 57.7%, followed by A3 (82, 24.9%), A5 (52, 15.8%), SE (4, 1.2%), and AF (1, 0.3%) (Figure 5B). In addition, the results of the analysis indicated that only a single isoform was detected for 100 (1.36%) unigenes. Two, three and four isoforms were found for 2,481 (33.85%), 1,705 (23.26%), and 1,074 (14.65%) genes, respectively. 304 (4.15%) genes were detected for more than ten splice isoforms (Figure 5C).

### 3.3 Functional annotation of transcripts

**FIGURE 6:**
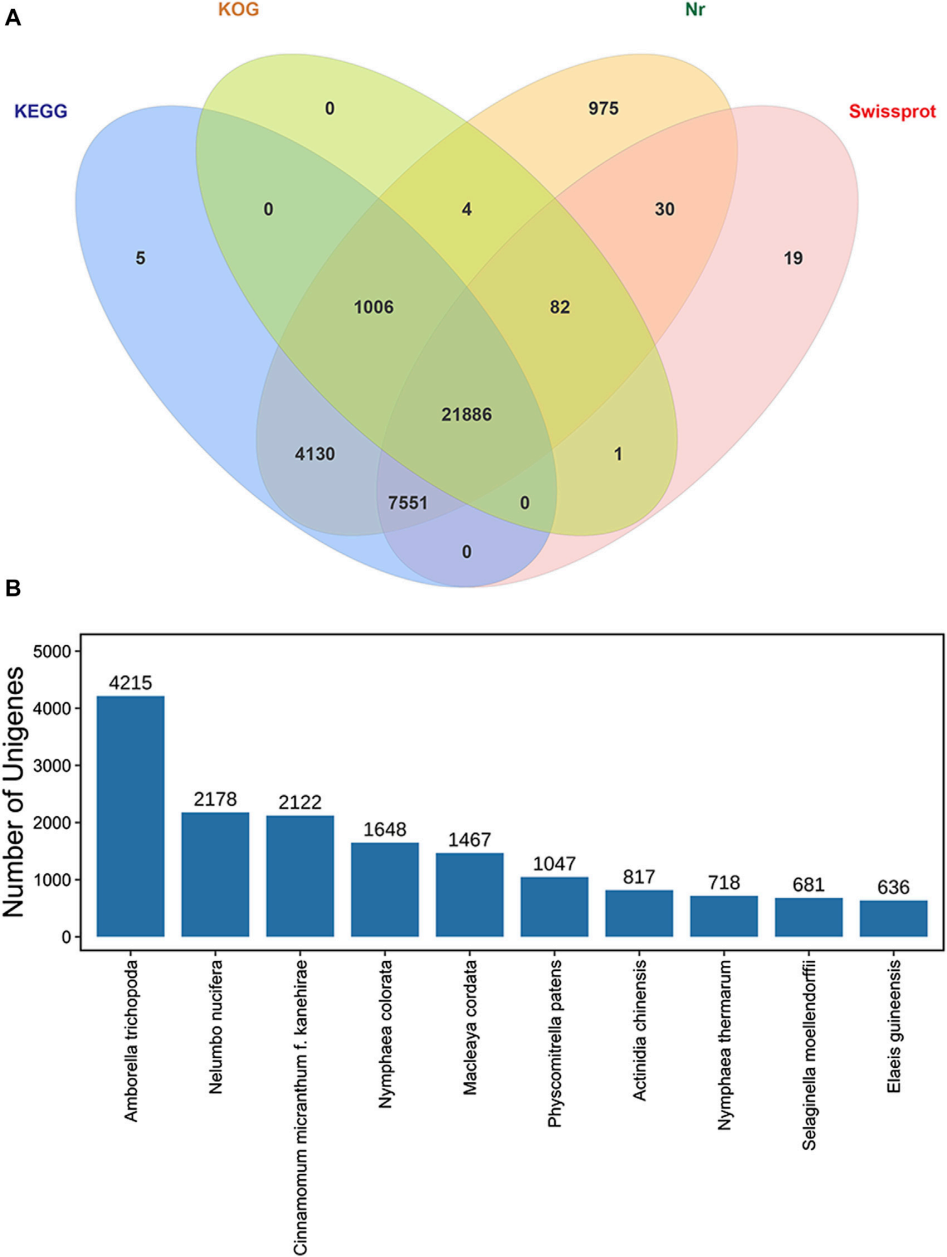
Functional annotation of isoforms in *P. orientalis*. **(A)** Venn diagram of annotated transcripts in the NCBI nr, Swiss-Prot, KOG, and KEGG databases. **(B)** The top ten nr homologous species of transcripts compared to *P. orientalis*.

All 37,111 transcripts were functionally annotated by comparing the nr, Swiss-Prot, KOG, and KEGG databases, and totally 35,689 transcripts (96.17%) were annotated by PacBio in at least one database (Figure 6A). Of these, we annotated 35,664 (96.10%), 34,578 (93.17%), 22,979 (61.92%) and 29,569 (79.68%) genes in the NCBI nr, KEGG, KOG and Swiss-Prot databases, respectively. In addition, 21,886 (58.97%) transcripts were significantly correlated with sequences in the four databases, while a total of 1,422 (3.83%) transcripts was not available for functional annotation and might be novel genes in the *P. orientalis* transcriptome (Table 1).

**TABLE 1:** Functional annotation of transcripts.

Database	Annotated transcripts	Percent (%)
NCBI nr	35,664	96.10
KEGG	34,578	93.17
KOG	22,979	61.92
Swiss-Prot	29,569	79.68
In all databases	21,886	58.97
In at least one database	35,689	96.17
Without annotation	1,422	3.83
All transcripts	37,111	100

According to homologous gene analysis, the results showed that the species with the most matching transcripts belonged to *Amborella trichopoda* (4,215, 11.36%), *Nelumbo nucifera* (2,178, 5.87%), *Cinnamomum micranthum* f. *kanehirae* (2,122, 5.72%), *Nymphaea colorata* (1,648, 4.44%), *Macleaya cordata* (1,467, 3.95%), *Physcomitrella patens* (1,047, 2.82%), *Actinidia chinensis* (817, 2.20%), *Nymphaea thermarum* (718, 1.93%), *Selaginella moellendorffii* (681, 1.84%), and *Elaeis guineensis* (636, 1.71%). Of these, 74 species were found to be gymnosperms (Supplementary Table S1), and the highest 10 transcripts were found in *Selaginella moellendorffii* (681), *Platycladus orientalis* (424), *Ginkgo biloba* (394), T*axus chinensis* (389), *Pinus taeda* (349), *Pinus tabuliformis* (330), *Picea sitchensis* (315), *Pinus pinaster* (296), *Cunninghamia lanceolate* (232), and *Picea abies* (190) (Figure 6B).

### 3.4 KOG and GO annotation

**FIGURE 7:**
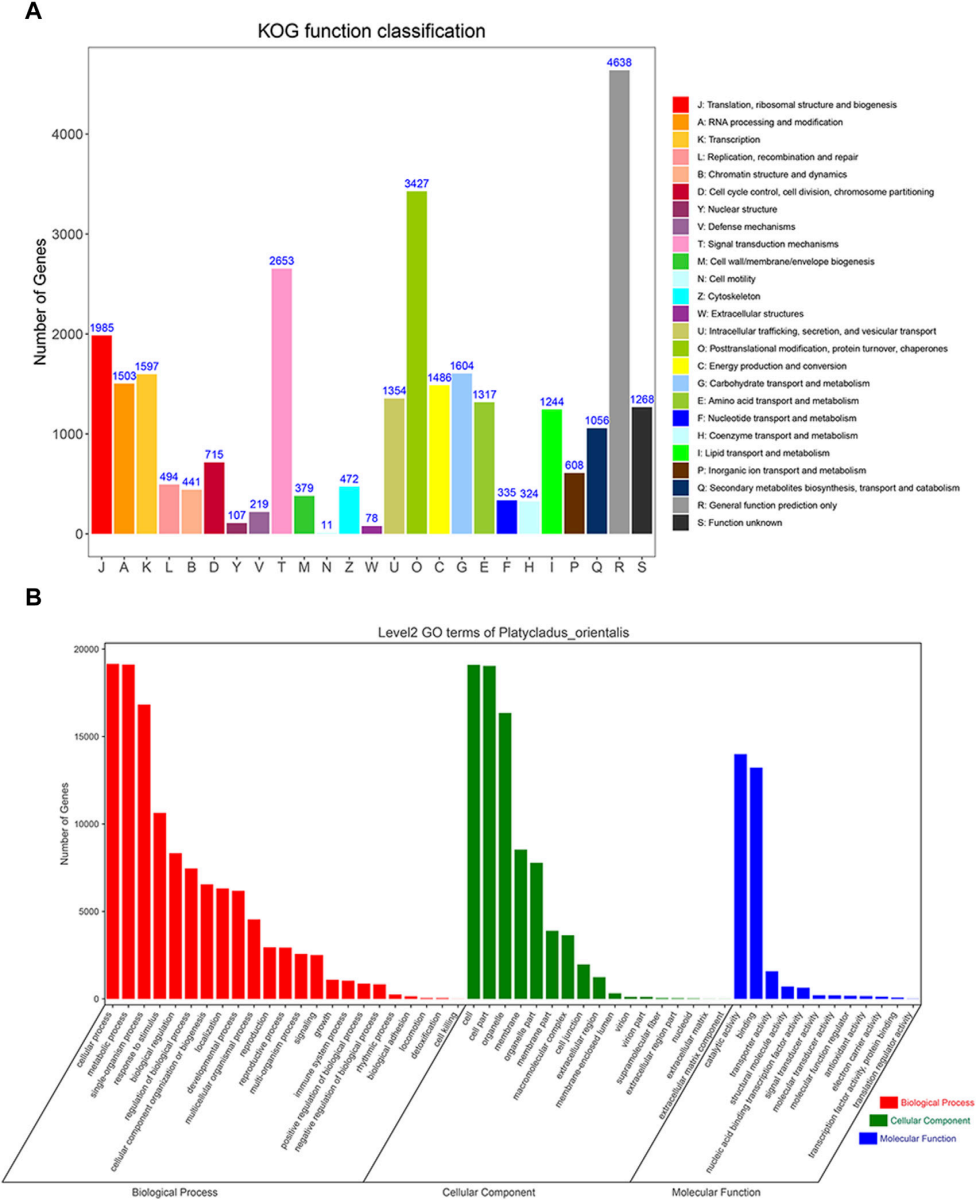
KOG and GO enrichment analyses of *P. orientalis* transcripts. **(A)** KOG annotation of transcript sequences. **(B)** Gene Ontology term classifications of transcript sequences.

KOG analysis showed that the transcript isoforms could be divided into 25 groups (Figure 7A). Among them, the number of general function prediction only transcripts (Group R) was the highest (4,638), followed by Group O (posttranslational modification, protein turnover, chaperones; 3,427) and Group T (signal transduction mechanisms; 2,653). Cell motility (Group N, 11) had the fewest transcripts, and there were 1,268 transcripts with unknown functions. Furthermore, GO analysis showed that all 37,111 transcripts were enriched in the biological process (BP), cellular component (CC), and molecular function (MF) categories, containing 52 subgroups (Figure 7B). Transcripts in the biological process category were mainly enriched for cellular, metabolic, single-organism, and other processes. Transcripts involved in the cellular component category were mainly associated with the cell, cell part, organelle, membrane, and organelle part. The molecular function category mainly included catalytic activity, binding, transporter activity and structural molecular activity, which indicating active metabolic processes existing in *P. orientalis*.

### 3.5 Analysis of KEGG pathway annotation

**FIGURE 8:**
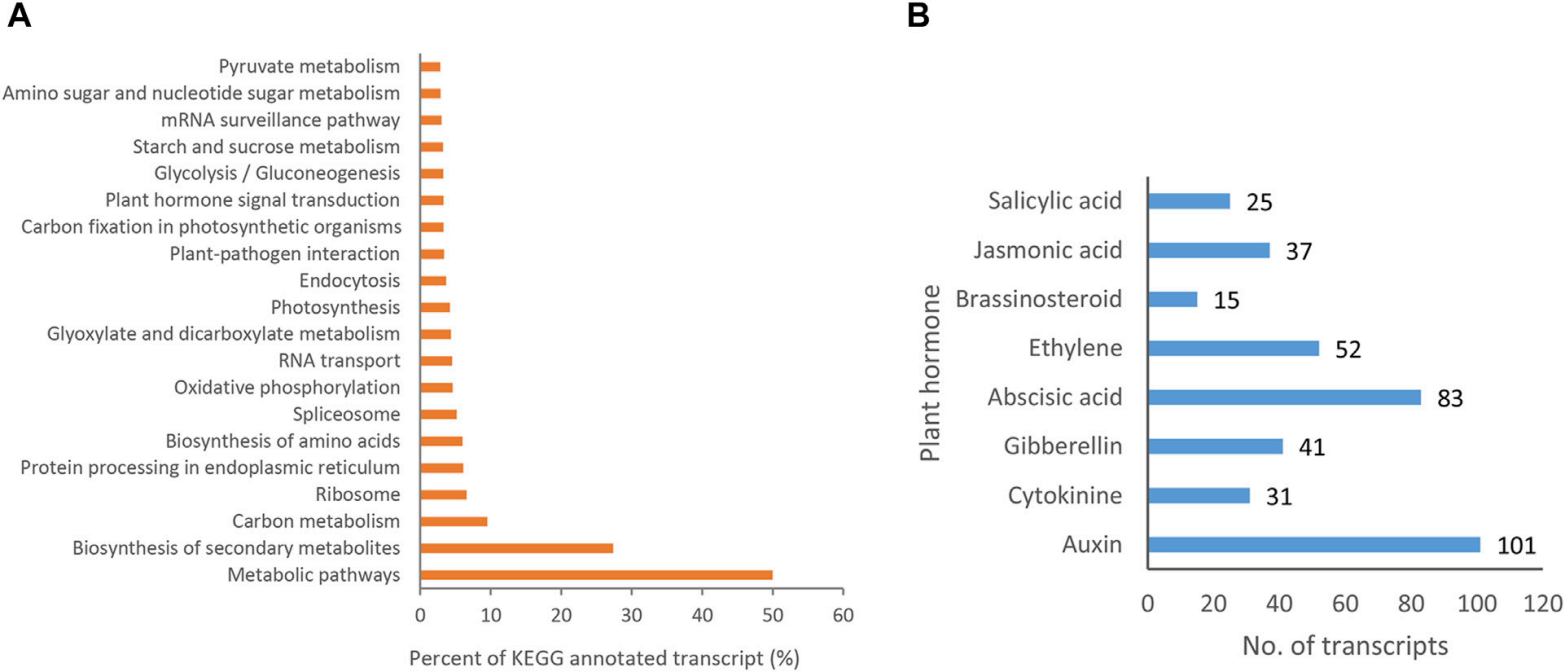
KEGG pathway annotation. **(A)** Top 20 KEGG metabolic pathways with the most transcript numbers. **(B)** Transcripts involved in plant hormone signal transduction pathways.

The KEGG analysis showed that 11,665 (31.43%) transcript isoforms were assigned to 136 KEGG pathways in *P. orientalis* (Supplementary Table S2). The functional pathways were first divided into five KEGG pathway categories, including cellular processes (4), environmental information processing (4), genetic information processing (21), metabolism (105), and organismal systems (2). Among them, “Metabolism” was the largest group, containing 105 (77.2%) pathways. In addition, the greatest number of pathways involving in special orthogroups were metabolic pathways (5,831, 49.99%), followed by biosynthesis of secondary metabolites (3,194, 27.38%), carbon metabolism (1,111, 9.52%) and biosynthesis of amino acids (703, 6.57%). The top 20 KEGG metabolic pathways with the most transcript numbers were shown in Figure 8A. These results provided large amount of data for exploration of the genetic resources of *P. orientalis*.

Plant hormone signal played an important role in regulating plant growth, flowering, and development process. A total of 385 transcript isoforms for 8 types of hormones were detected to be contained in plant hormone signal transduction pathways in *P. orientalis* (ko04075; Figure 8B; Supplementary Table S3). Among them, the number of transcripts associated with auxin was the largest (101, 26.23%), followed by abscisic acid (83, 21.56%) and ethylene (52, 13.51%). However, the number of transcripts associated with brassinosteroids was the lowest (15, 3.90%). 101, 31, 41, 83, 52, 15, 37, 25 transcripts were annotated in the Auxin, cytokinine, gibberellin, abscisic acid, Ethylene, Brassinosteroid, Jasmonic acid, and salicylic acid pathways, respectively. These mainly encoded 35 key genes in the plant hormone signal transduction pathway in *P. orientalis* (Supplementary Table S3). Of these, auxin/indole-3-acetic acid protein (AUX/IAA; 67), abscisic acid receptor PYR/PYL family (PYL, 30), serine/threonine-protein kinase SRK2 (SNRK2, 20) owned the most transcripts. These results provided information for studying the development of *P. orientalis*.

## 4 Discussion

In this study, the third-generation high-throughput sequencing technology represented by single-molecule real-time sequencing based on PacBio plaform (Liao et al., 2015), combined with sequence and bioinformatics analysis, was used to produce the full-length transcriptome sequence of *P. orientalis*. The target sequence can be read directly, without PCR amplification, by using the instrument, which is widely used in transcriptome sequencing in species without reference genomes (Ren et al., 2016). In this study, a total of 37,111 high-quality isoforms were obtained after redundancy removal and error correction, with an average length of 1,999 bp and an N50 of 2,317 nt, indicating long read lengths and high continuity of third-generation sequencing. Comparison against the nr database identified 35,664 unigenes in 480 species, accounting for 96.1%, among which the sequences were most similar to those of *A*. *trichopoda*, followed by *N. nucifera* and *C. micranthum* f. *kanehirae*. However, 228,948 transcripts ≥200 nt were obtained by second-generation sequencing using *de novo* in *P. orientalis*. The mean length was 686 nt and the N50 was 1,320 nt, according to Hu et al. (2016). The average length of the unigenes assembled by second-generation sequencing was 762.6 bp, and the value of N50 was 1,428 nt in another study in *P. orientalis* (Dong et al., 2023). The results showed that the quality, length and annotated gene information of third-generation sequencing were better than those in second-generation sequencing. In the present study, according to the notes in the nr database, most genes were annotated to *A*. *trichopoda*, and there was almost no obvious genetic relationship with any other angiosperm. Studies have shown that *A*. *trichopoda* may be the most primitive of angiosperms, indicating that *P. orientalis* may also be an ancient species. With the deepening of biological research on *P.* orientalis, an increasing number of genes are registered in GenBank, and it is expected that more gene annotations and functions will be analyzed.

As a raw material for the extraction of spices and essential oils, a large number of transcripts from *P. orientalis* were associated with secondary metabolite synthesis, transport and metabolism (1,056), amino acid transport and metabolism (1,317). The formation of spices was closely related to the contents and synthesis of terpenes. Among them, biosynthesis of secondary metabolites accounted for 3,194 unigenes (27.38%), biosynthesis of amino acids accounted for 703 unigenes (6.03%), and terpenoid backbone biosynthesis accounted for 157 unigenes (1.35%) (Supplementary Table S2). The signal transduction mechanisms (Group T) was the third most abundant category, indicating the complexity of various regulatory mechanisms in *P. orientalis*. These findings laid a foundation for the determination of fragrance quality in *P. orientalis*.

As codominant genetic markers with a strong specificity, good repeatability and high polymorphism, SSRs have been widely used in genetic diversity detection, quantitative trait loci (QTL) mapping, genetic map construction, genotype analysis, the origin and evolution of plants in and near species. They have strong application potential in the field of molecular breeding (Liu et al., 2012). In this study, we analyzed the full-length transcriptome of *P. orientalis* and identified unigenes from single-nucleotide to six-nucleotide repeat motif SSR loci. A total of 5,645 SSR loci were obtained. The frequencies of dinucleotide and trinucleotide repeats were 29.6% and 52.7% among the total SSR loci, respectively. However, 5,296 SSRs were identified through second-generation sequencing, and dinucleotide repeats were most abundant, accounting for 70.31% (1,279), followed by trinucleotide (522, 28.70%) and tetranucleotide (16, 0.88%) repeat motifs in *P. orientalis* (Hu et al., 2016). This provides a basis for future development of SSR primers and lays a good foundation for further development of integrating SSR markers to increase the density of a linkage map.

In addition to coding RNAs, the effects of non-coding RNAs on plant growth and development have recently been explored gradually (Wang et al., 2016). Unlike coding RNAs, lncRNAs are not directly homologous among related species. Thus, information from one species is of few reference value in lncRNA predictions for other species (Hoang et al., 2017). LncRNAs play important regulatory roles in biological processes such as nutrient metabolism, male sterility, plant flowering, organogenesis and leaf senescence (Chekanova, 2015; Meng et al., 2018; Bouba et al., 2019; Sun et al., 2020; Zhang et al., 2020). Thanks to the fast development of high-throughput sequencing technology, biotechnology and bioinformatics, large amount of lncRNAs have been detected in plants such as *Medicago truncatula* (Wen et al., 2007), *Arabidopsis thaliana* (Ben et al., 2009), wheat (Xin et al., 2011) and *Zea mays* (Boerner and McGinnis, 2017). To predict lncRNAs of *P. orientalis*, CPC and CNCI were used. A total of 1,201 lncRNAs were predicted, which were mostly concentrated in short transcripts. The results of this study can provide a reference for future lncRNA function exploration.

Transcription factors play regulatory roles in plant abiotic stress, pigment accumulation, and flower formation. For example, the bZIP transcription factor plays a role in the vegetable response to low-temperature stress (Xu et al., 2023), and the MYB transcription factor regulates pigment formation and accumulation processes by regulating gene expression. A total of 51 transcription factor families and 1,659 transcription factors were predicted in the present study, among which 11 families were related to hormone metabolism and flowering regulation, including the *bHLH, GRAS, MYB, ERF, NAC, ARF, WRKY, MIKE, GATA, TCP*, and *AP2* families. The *ARR* family has been implicated in sex determination in *Polulus* species (Müller et al., 2020; Xue et al., 2020). The identification of these transcription factors can provide important sequence resources for subsequent cones development and sex differentiation gene cloning, bioinformatics analysis and gene function verification.

## 5 Conclusion

In summary, this study successfully assembled and obtained the full-length transcriptome of *P. orientalis* using SMRT sequencing and conducted basic sequence analysis of the transcript sequences, as well as KOG classification, GO analysis, pathway enrichment analysis and functional prediction of unigenes. In addition, CDSs, SSRs, TFs, lncRNAs and AS events were predicted. The results enriched genetic information of *P. orientalis*, provided a reference transcriptome for second-generation sequencing of differentially expressed genes, and laid a solid foundation for further functional gene mining, molecular biology research and breeding in *P. orientalis*.

## Data Availability

The original contributions presented in the study are publicly available. This data can be found here: NCBI Sequence Read Archives (SRA) database, with the BioProject ID: PRJNA1043448, BioSample: SAMN38341079: Po (TaxID: 58046) and SRA: SRR26896759. The repository and accession number can be found at https://www.ncbi.nlm.nih.gov/sra.
