# Supplementary material for: Full-length transcriptome characterization of Platycladus orientalis based on the PacBio platform
Source: Front Genet. 2024 Jan 18;15:1345039. doi: 10.3389/fgene.2024.1345039 (PMC10830785; doi:10.3389/fgene.2024.1345039)
Supplement: Supplementary file 1 [file Table3.DOC]

Supplementary Table S3 Transcript isoforms involved in Plant hormone signal transduction pathway in *Platycladus orentalis*

| Hormone | K_ID | Transcripts | No. of transcripts | Description |
| --- | --- | --- | --- | --- |
| Auxin | K13946 | Isoform0008922, Isoform0010659, Isoform0010902, Isoform0014342, Isoform0014846, Isoform0017282, Isoform0019216, Isoform0019632, Isoform0022962, Isoform0024725 | 10 | AUX1, LAX; auxin influx carrier (AUX1 LAX family) |
| Auxin | K14484 | Isoform0004794, Isoform0004996, Isoform0005018, Isoform0005231, Isoform0005473, Isoform0005745, Isoform0006368, Isoform0006559, Isoform0006862, Isoform0006961, Isoform0007060, Isoform0007274, Isoform0007524, Isoform0007537, Isoform0008074, Isoform0008123, Isoform0008278, Isoform0008358, Isoform0008403, Isoform0008434, Isoform0008446, Isoform0008826, Isoform0009255, Isoform0009495, Isoform0010038, Isoform0010117, Isoform0010287, Isoform0010295, Isoform0010357, Isoform0010715, Isoform0011223, Isoform0011346, Isoform0011971, Isoform0012035, Isoform0012237, Isoform0012892, Isoform0013223, Isoform0013444, Isoform0013571, Isoform0014041, Isoform0015058, Isoform0015135, Isoform0015462, Isoform0015590, Isoform0016643, Isoform0016884, Isoform0016902, Isoform0017028, Isoform0017201, Isoform0018065, Isoform0018382, Isoform0018732, Isoform0020476, Isoform0021304, Isoform0021454, Isoform0021471, Isoform0021734, Isoform0022652, Isoform0023253, Isoform0024123, Isoform0024314, Isoform0024329, Isoform0024971, Isoform0027778, Isoform0028132, Isoform0032511, Isoform0033390 | 67 | IAA; auxin-responsive protein IAA |
| Auxin | K14485 | Isoform0003380, Isoform0004287, Isoform0008650, Isoform0012249, Isoform0013002 | 5 | TIR1; transport inhibitor response 1 |
| Auxin | K14486 | Isoform0001777, Isoform0001993, Isoform0001998, Isoform0002425 | 4 | ARF; auxin response factor |
| Auxin | K14487 | Isoform0007754, Isoform0010198, Isoform0011122, Isoform0011556, Isoform0012507, Isoform0012633, Isoform0015668, Isoform0016404, Isoform0022351, Isoform0034399 | 10 | GH3; auxin responsive GH3 gene family |
| Auxin | K14488 | Isoform0028483, Isoform0029714, Isoform0029977, Isoform0031965, Isoform0034909 | 5 | SAUR; SAUR family protein |
| Cytokinine | K14491 | Isoform0001164, Isoform0002275, Isoform0002537, Isoform0003620, Isoform0003664, Isoform0004293, Isoform0004717, Isoform0005318, Isoform0005811, Isoform0005842, Isoform0005974, Isoform0006135, Isoform0007285, Isoform0007875, Isoform0008657, Isoform0010610, Isoform0011243, Isoform0012336, Isoform0013661 | 19 | ARR-B; two-component response regulator ARR-B family |
| Cytokinine | K14492 | Isoform0025130 | 1 | ARR-A; two-component response regulator ARR-A family |
| Cytokinine | K14490 | Isoform0030691, Isoform0030828, Isoform0031534, Isoform0032150, Isoform0034221 | 5 | AHP; histidine-containing phosphotransfer peotein |
| Cytokinine | K14489 | Isoform0000557, Isoform0000945, Isoform0001192, Isoform0001400, Isoform0001982, Isoform0003027 | 6 | AHK2_3_4; CRE, arabidopsis histidine kinase 2/3/4 (cytokinin receptor) [EC:2.7.13.3] |
| Gibberellin | K14493 | Isoform0014277, Isoform0015996, Isoform0016013, Isoform0016123, Isoform0016359, Isoform0018139, Isoform0020641, Isoform0023032, Isoform0025606, Isoform0030860 | 10 | GID1; gibberellin receptor GID1 [EC:3.-.-.-] |
| Gibberellin | K14494 | Isoform0004855, Isoform0005789, Isoform0006614, Isoform0006843, Isoform0009620, Isoform0011426, Isoform0011837, Isoform0012156, Isoform0012543, Isoform0012759, Isoform0012935, Isoform0013412, Isoform0014340, Isoform0014427, Isoform0016237, Isoform0017479, Isoform0022950, Isoform0026043 | 18 | DELLA; DELLA protein |
| Gibberellin | K14495 | Isoform0018924, Isoform0022800, Isoform0032792 | 3 | GID2, SLY1; F-box protein GID2 |
| Gibberellin | K12126 | Isoform0002848, Isoform0003989, Isoform0004601, Isoform0005507, Isoform0006477, Isoform0007411, Isoform0016613, Isoform0023224, Isoform0028861, Isoform0031123 | 10 | PIF3; phytochrome-interacting factor 3 |
| Abscisic acid | K14432 | Isoform0002698, Isoform0003754, Isoform0005490, Isoform0008508, Isoform0009836, Isoform0010265, Isoform0018486, Isoform0019160, Isoform0020048, Isoform0020459, Isoform0021253, Isoform0023272, Isoform0023440, Isoform0033216 | 14 | ABF; ABA responsive element binding factor |
| Abscisic acid | K14496 | Isoform0010343, Isoform0011057, Isoform0011215, Isoform0013874, Isoform0015203, Isoform0015461, Isoform0015860, Isoform0016103, Isoform0016683, Isoform0018440, Isoform0020055, Isoform0024371, Isoform0025231, Isoform0025445, Isoform0025739, Isoform0027147, Isoform0027449, Isoform0027470, Isoform0027633, Isoform0027732, Isoform0029448, Isoform0029661, Isoform0029985, Isoform0030782, Isoform0030855, Isoform0032031, Isoform0032428, Isoform0033040, Isoform0035610, Isoform0035790 | 30 | PYL; abscisic acid receptor PYR/PYL family |
| Abscisic acid | K14497 | Isoform0002678, Isoform0003253, Isoform0004204, Isoform0005202, Isoform0005692, Isoform0006085, Isoform0006561, Isoform0007501, Isoform0007669, Isoform0007986, Isoform0008782, Isoform0009851, Isoform0012062, Isoform0012283, Isoform0017295, Isoform0018636, Isoform0021498, Isoform0026973, Isoform0031420 | 19 | PP2C; protein phosphatase 2C [EC:3.1.3.16] |
| Abscisic acid | K14498 | Isoform0011982, Isoform0013821, Isoform0015559, Isoform0015900, Isoform0016059, Isoform0017079, Isoform0018646, Isoform0018776, Isoform0018856, Isoform0019890, Isoform0020554, Isoform0020719, Isoform0021520, Isoform0022453, Isoform0023064, Isoform0023329, Isoform0024183, Isoform0027527, Isoform0027754, Isoform0029786 | 20 | SNRK2; serine/threonine-protein kinase SRK2 [EC:2.7.11.1] |
| Ethylene | K14509 | Isoform0002893, Isoform0003112, Isoform0004126, Isoform0004511, Isoform0004616, Isoform0004624, Isoform0005053, Isoform0005721 | 8 | ETR, ERS; ethylene receptor [EC:2.7.13.-] |
| Ethylene | K14510 | Isoform0000563, Isoform0000773, Isoform0000812, Isoform0000818, Isoform0005127 | 5 | CTR1; serine/threonine-protein kinase CTR1 [EC:2.7.11.1] |
| Ethylene | K14512 | Isoform0015495, Isoform0019810, Isoform0022362, Isoform0023883, Isoform0024898, Isoform0026935 | 6 | MPK6; mitogen-activated protein kinase 6 [EC:2.7.11.24] |
| Ethylene | K14513 | Isoform0001321, Isoform0010521 | 2 | EIN2; ethylene-insensitive protein 2 |
| Ethylene | K14514 | Isoform0002492, Isoform0003170, Isoform0003875, Isoform0003993, Isoform0004407, Isoform0004438, Isoform0004834, Isoform0008015, Isoform0009120, Isoform0010920, Isoform0012956, Isoform0014346, Isoform0016146, Isoform0020096, Isoform0021088, Isoform0028064 | 16 | EIN3; ethylene-insensitive protein 3 |
| Ethylene | K14515 | Isoform0004171, Isoform0004246, Isoform0004423, Isoform0004658, Isoform0005074, Isoform0005826, Isoform0005978, Isoform0006422, Isoform0006510, Isoform0009251, Isoform0013479, Isoform0014896, Isoform0023037, Isoform0025603, Isoform0027430 | 15 | EBF1_2; EIN3-binding F-box protein |
| Brassinosteroid | K13415 | Isoform0009405, Isoform0026232 | 2 | BRI1; protein brassinosteroid insensitive 1 [EC:2.7.10.1 2.7.11.1] |
| Brassinosteroid | K14500 | Isoform0007624, Isoform0008656, Isoform0008776, Isoform0009751, Isoform0012111, Isoform0014551, Isoform0017325, Isoform0017464, Isoform0023234, Isoform0023813 | 10 | BSK; BR-signaling kinase [EC:2.7.11.1] |
| Brassinosteroid | K14504 | Isoform0031325 | 1 | TCH4; xyloglucan: xyloglucosyl transferase TCH4 [EC:2.4.1.207] |
| Brassinosteroid | K14505 | Isoform0017840, Isoform0020755 | 2 | CYCD3; cyclin D3, plant |
| Jasmonic acid | K13422 | Isoform0003172, Isoform0003463, Isoform0004055, Isoform0004841, Isoform0005635, Isoform0005767, Isoform0009868, Isoform0011574 | 8 | MYC2; transcription factor MYC2 |
| Jasmonic acid | K13463 | Isoform0005700, Isoform0007288, Isoform0007328, Isoform0007968, Isoform0008848, Isoform0009363, Isoform0010003, Isoform0010431, Isoform0010728, Isoform0014469, Isoform0016755, Isoform0026270 | 12 | COI-1; coronatine-insensitive protein 1 |
| Jasmonic acid | K13464 | Isoform0005791, Isoform0007146, Isoform0008034, Isoform0009665, Isoform0018672, Isoform0018881, Isoform0020068, Isoform0023425, Isoform0023575, Isoform0025293, Isoform0027057, Isoform0031350, Isoform0032963, Isoform0033177 | 14 | JAZ; jasmonate ZIM domain-containing protein |
| Jasmonic acid | K14506 | Isoform0009668, Isoform0010436, Isoform0030507 | 3 | JAR1_4_6; jasmonic acid-amino synthetase [EC:6.3.2.52] |
| Salicylic acid | K13449 | Isoform0034562, Isoform0035279 | 2 | PR1; pathogenesis-related protein 1 |
| Salicylic acid | K14431 | Isoform0005243, Isoform0005506, Isoform0005586, Isoform0007801, Isoform0008326, Isoform0010592, Isoform0010745, Isoform0011517, Isoform0014241, Isoform0015016, Isoform0015935, Isoform0016436, Isoform0019445 | 13 | TGA; transcription factor TGA |
| Salicylic acid | K14508 | Isoform0003828, Isoform0007656, Isoform0010619, Isoform0010946, Isoform0011076, Isoform0011541, Isoform0011897, Isoform0014351, Isoform0017471, Isoform0022040 | 10 | NPR1; regulatory protein NPR1 |
