# Supplementary material for: Full-length transcriptome characterization of Platycladus orientalis based on the PacBio platform
Source: Front Genet. 2024 Jan 18;15:1345039. doi: 10.3389/fgene.2024.1345039 (PMC10830785; doi:10.3389/fgene.2024.1345039)
Supplement: Supplementary file 2 [file Table1.DOC]

Supplementary Table S1 Nr homologous species of transcripts in gymnosperm

| Species | Number | Species | Number |
| --- | --- | --- | --- |
| *Selaginella moellendorffii* | 681 | *Thujopsis dolabrata* | 7 |
| *Platycladus orientalis* | 424 | *Chamaecyparis formosensis* | 7 |
| *Ginkgo biloba* | 394 | *Chamaecyparis hodginsii* | 6 |
| *Taxus chinensis* | 389 | *Cephalotaxus hainanensis* | 6 |
| *Pinus taeda* | 349 | *Pinus mugo* | 6 |
| *Pinus tabuliformis* | 330 | *Juniperus oxycedrus* | 4 |
| *Picea sitchensis* | 315 | *Juniperus microsperma* | 4 |
| *Pinus pinaster* | 296 | *Taxus mairei* | 4 |
| *Cunninghamia lanceolata* | 232 | *Juniperus phoenicea* | 4 |
| *Picea abies* | 190 | *Cupressus chengiana* | 4 |
| *Cryptomeria japonica* | 184 | *Juniperus cedrus* | 3 |
| *Pinus massoniana* | 145 | *Juniperus saltuaria* | 3 |
| *Pinus radiata* | 109 | *Cupressus torulosa* | 3 |
| *Pinus sylvestris* | 101 | *Juniperus bermudiana* | 3 |
| *Larix kaempferi* | 98 | *Picea engelmannii x Picea glauca* | 3 |
| *Thuja plicata* | 55 | *Juniperus rigida* | 3 |
| *Pinus lambertiana* | 48 | *Cupressus gigantea* | 3 |
| *Picea mariana* | 47 | *Podocarpus rubens* | 3 |
| *Juniperus communis* | 44 | *Abies magnifica* | 3 |
| *Pinus koraiensis* | 28 | *Pinus armandii* | 2 |
| *Thuja occidentalis* | 27 | *Juniperus flaccida* | 2 |
| *Sequoia sempervirens* | 26 | *Pinus sibirica* | 1 |
| *Larix gmelinii* | 21 | *Pinus canariensis* | 1 |
| *Pinus elliottii* | 20 | *Picea breweriana* | 1 |
| *Thuja koraiensis* | 19 | *Picea engelmannii* | 1 |
| *Picea wilsonii* | 19 | *Juniperus blancoi* | 1 |
| *Cupressus sempervirens* | 16 | *Juniperus tibetica* | 1 |
| *Pinus contorta* | 14 | *Juniperus przewalskii* | 1 |
| *Juniperus monosperma* | 13 | *Thuja sutchuenensis* | 1 |
| *Picea likiangensis* | 12 | *Picea smithiana* | 1 |
| *Larix laricina* | 10 | *Juniperus virginiana* | 1 |
| *Pinus pumila* | 9 | *Picea asperata* | 1 |
| *Picea rubens* | 9 | *Abies holophylla* | 1 |
| *Pinus kesiya* | 9 | *Pinus resinosa* | 1 |
| *Pinus banksiana* | 8 | *Juniperus procumbens* | 1 |
| *Abies alba* | 8 | *Juniperus scopulorum* | 1 |
| *Pinus monticola* | 7 | *Picea koyamae* | 1 |
