# Supplementary material for: Full-length transcriptome characterization of Platycladus orientalis based on the PacBio platform
Source: Front Genet. 2024 Jan 18;15:1345039. doi: 10.3389/fgene.2024.1345039 (PMC10830785; doi:10.3389/fgene.2024.1345039)
Supplement: Supplementary file 3 [file Table2.DOC]

Supplementary Table S2 KEGG metabolic pathways involved in *Platycladus orentalis*

| KEGG_A_class | Pathway | Count (11665) | Ratio (%) | Pathway ID |
| --- | --- | --- | --- | --- |
| Cellular Processes | Endocytosis | 427 | 3.66 | ko04144 |
|  | Peroxisome | 270 | 2.31 | ko04146 |
|  | Phagosome | 240 | 2.06 | ko04145 |
|  | Autophagy - other eukaryotes | 78 | 0.67 | ko04136 |
| Environmental Information Processing | Plant hormone signal transduction | 385 | 3.30 | ko04075 |
|  | MAPK signaling pathway - plant | 278 | 2.38 | ko04016 |
|  | Phosphatidylinositol signaling system | 103 | 0.88 | ko04070 |
|  | ABC transporters | 58 | 0.50 | ko02010 |
| Genetic Information Processing | Ribosome | 766 | 6.57 | ko03010 |
|  | Protein processing in endoplasmic reticulum | 714 | 6.12 | ko04141 |
|  | Spliceosome | 605 | 5.19 | ko03040 |
|  | RNA transport | 531 | 4.55 | ko03013 |
|  | mRNA surveillance pathway | 354 | 3.03 | ko03015 |
|  | Ubiquitin mediated proteolysis | 261 | 2.24 | ko04120 |
|  | RNA degradation | 228 | 1.95 | ko03018 |
|  | Proteasome | 177 | 1.52 | ko03050 |
|  | Aminoacyl-tRNA biosynthesis | 159 | 1.36 | ko00970 |
|  | Ribosome biogenesis in eukaryotes | 153 | 1.31 | ko03008 |
|  | RNA polymerase | 114 | 0.98 | ko03020 |
|  | Protein export | 98 | 0.84 | ko03060 |
|  | Nucleotide excision repair | 83 | 0.71 | ko03420 |
|  | DNA replication | 61 | 0.52 | ko03030 |
|  | Base excision repair | 57 | 0.49 | ko03410 |
|  | Basal transcription factors | 55 | 0.47 | ko03022 |
|  | Mismatch repair | 49 | 0.42 | ko03430 |
|  | Homologous recombination | 46 | 0.39 | ko03440 |
|  | SNARE interactions in vesicular transport | 46 | 0.39 | ko04130 |
|  | Sulfur relay system | 14 | 0.12 | ko04122 |
|  | Non-homologous end-joining | 10 | 0.09 | ko03450 |
| Metabolism | Metabolic pathways | 5831 | 49.99 | ko01100 |
|  | Biosynthesis of secondary metabolites | 3194 | 27.38 | ko01110 |
|  | Carbon metabolism | 1111 | 9.52 | ko01200 |
|  | Biosynthesis of amino acids | 703 | 6.03 | ko01230 |
|  | Oxidative phosphorylation | 536 | 4.59 | ko00190 |
|  | Glyoxylate and dicarboxylate metabolism | 509 | 4.36 | ko00630 |
|  | Photosynthesis | 492 | 4.22 | ko00195 |
|  | Carbon fixation in photosynthetic organisms | 387 | 3.32 | ko00710 |
|  | Glycolysis / Gluconeogenesis | 381 | 3.27 | ko00010 |
|  | Starch and sucrose metabolism | 377 | 3.23 | ko00500 |
|  | Amino sugar and nucleotide sugar metabolism | 338 | 2.90 | ko00520 |
|  | Pyruvate metabolism | 333 | 2.85 | ko00620 |
|  | Fatty acid metabolism | 282 | 2.42 | ko01212 |
|  | Cysteine and methionine metabolism | 276 | 2.37 | ko00270 |
|  | Glycine, serine and threonine metabolism | 274 | 2.35 | ko00260 |
|  | Phenylpropanoid biosynthesis | 243 | 2.08 | ko00940 |
|  | Citrate cycle (TCA cycle) | 216 | 1.85 | ko00020 |
|  | Purine metabolism | 207 | 1.77 | ko00230 |
|  | Fatty acid biosynthesis | 197 | 1.69 | ko00061 |
|  | Glycerophospholipid metabolism | 194 | 1.66 | ko00564 |
|  | Pentose phosphate pathway | 190 | 1.63 | ko00030 |
|  | Fructose and mannose metabolism | 190 | 1.63 | ko00051 |
|  | Glutathione metabolism | 184 | 1.58 | ko00480 |
|  | Porphyrin and chlorophyll metabolism | 165 | 1.41 | ko00860 |
|  | Alanine, aspartate and glutamate metabolism | 163 | 1.40 | ko00250 |
|  | Glycerolipid metabolism | 162 | 1.39 | ko00561 |
|  | Flavonoid biosynthesis | 161 | 1.38 | ko00941 |
|  | Propanoate metabolism | 159 | 1.36 | ko00640 |
|  | Terpenoid backbone biosynthesis | 157 | 1.35 | ko00900 |
|  | Fatty acid degradation | 155 | 1.33 | ko00071 |
|  | 2-Oxocarboxylic acid metabolism | 146 | 1.25 | ko01210 |
|  | alpha-Linolenic acid metabolism | 134 | 1.15 | ko00592 |
|  | Inositol phosphate metabolism | 132 | 1.13 | ko00562 |
|  | Arginine and proline metabolism | 129 | 1.11 | ko00330 |
|  | Galactose metabolism | 127 | 1.09 | ko00052 |
|  | Ascorbate and aldarate metabolism | 127 | 1.09 | ko00053 |
|  | Pentose and glucuronate interconversions | 123 | 1.05 | ko00040 |
|  | Nitrogen metabolism | 117 | 1.00 | ko00910 |
|  | Phenylalanine, tyrosine and tryptophan biosynthesis | 116 | 0.99 | ko00400 |
|  | Linoleic acid metabolism | 115 | 0.99 | ko00591 |
|  | Arginine biosynthesis | 113 | 0.97 | ko00220 |
|  | beta-Alanine metabolism | 113 | 0.97 | ko00410 |
|  | Tryptophan metabolism | 107 | 0.92 | ko00380 |
|  | Valine, leucine and isoleucine degradation | 106 | 0.91 | ko00280 |
|  | Pyrimidine metabolism | 100 | 0.86 | ko00240 |
|  | N-Glycan biosynthesis | 99 | 0.85 | ko00510 |
|  | Ubiquinone and other terpenoid-quinone biosynthesis | 98 | 0.84 | ko00130 |
|  | Photosynthesis - antenna proteins | 90 | 0.77 | ko00196 |
|  | Tyrosine metabolism | 89 | 0.76 | ko00350 |
|  | Various types of N-glycan biosynthesis | 86 | 0.74 | ko00513 |
|  | Phenylalanine metabolism | 84 | 0.72 | ko00360 |
|  | Thiamine metabolism | 83 | 0.71 | ko00730 |
|  | Carotenoid biosynthesis | 80 | 0.69 | ko00906 |
|  | Biosynthesis of unsaturated fatty acids | 79 | 0.68 | ko01040 |
|  | Cyanoamino acid metabolism | 78 | 0.67 | ko00460 |
|  | Steroid biosynthesis | 76 | 0.65 | ko00100 |
|  | Cutin, suberine and wax biosynthesis | 74 | 0.63 | ko00073 |
|  | Lysine degradation | 67 | 0.57 | ko00310 |
|  | Sphingolipid metabolism | 67 | 0.57 | ko00600 |
|  | One carbon pool by folate | 62 | 0.53 | ko00670 |
|  | Selenocompound metabolism | 60 | 0.51 | ko00450 |
|  | Sulfur metabolism | 60 | 0.51 | ko00920 |
|  | Pantothenate and CoA biosynthesis | 55 | 0.47 | ko00770 |
|  | Brassinosteroid biosynthesis | 53 | 0.45 | ko00905 |
|  | Ether lipid metabolism | 51 | 0.44 | ko00565 |
|  | Biotin metabolism | 49 | 0.42 | ko00780 |
|  | Fatty acid elongation | 45 | 0.39 | ko00062 |
|  | Nicotinate and nicotinamide metabolism | 45 | 0.39 | ko00760 |
|  | Tropane, piperidine and pyridine alkaloid biosynthesis | 45 | 0.39 | ko00960 |
|  | Valine, leucine and isoleucine biosynthesis | 43 | 0.37 | ko00290 |
|  | Butanoate metabolism | 42 | 0.36 | ko00650 |
|  | Stilbenoid, diarylheptanoid and gingerol biosynthesis | 42 | 0.36 | ko00945 |
|  | Histidine metabolism | 41 | 0.35 | ko00340 |
|  | Zeatin biosynthesis | 39 | 0.33 | ko00908 |
|  | Other glycan degradation | 38 | 0.33 | ko00511 |
|  | Isoquinoline alkaloid biosynthesis | 36 | 0.31 | ko00950 |
|  | Folate biosynthesis | 34 | 0.29 | ko00790 |
|  | Arachidonic acid metabolism | 31 | 0.27 | ko00590 |
|  | Other types of O-glycan biosynthesis | 29 | 0.25 | ko00514 |
|  | Vitamin B6 metabolism | 27 | 0.23 | ko00750 |
|  | Diterpenoid biosynthesis | 27 | 0.23 | ko00904 |
|  | Lysine biosynthesis | 23 | 0.20 | ko00300 |
|  | Glycosaminoglycan degradation | 23 | 0.20 | ko00531 |
|  | Flavone and flavonol biosynthesis | 22 | 0.19 | ko00944 |
|  | Phosphonate and phosphinate metabolism | 21 | 0.18 | ko00440 |
|  | Riboflavin metabolism | 21 | 0.18 | ko00740 |
|  | Monobactam biosynthesis | 20 | 0.17 | ko00261 |
|  | C5-Branched dibasic acid metabolism | 19 | 0.16 | ko00660 |
|  | Glycosylphosphatidylinositol(GPI)-anchor biosynthesis | 18 | 0.15 | ko00563 |
|  | Limonene and pinene degradation | 17 | 0.15 | ko00903 |
|  | Glycosphingolipid biosynthesis - globo and isoglobo series | 14 | 0.12 | ko00603 |
|  | Taurine and hypotaurine metabolism | 13 | 0.11 | ko00430 |
|  | Monoterpenoid biosynthesis | 13 | 0.11 | ko00902 |
|  | Sesquiterpenoid and triterpenoid biosynthesis | 13 | 0.11 | ko00909 |
|  | Aflatoxin biosynthesis | 10 | 0.09 | ko00254 |
|  | Caffeine metabolism | 9 | 0.08 | ko00232 |
|  | Biosynthesis of various secondary metabolites - part 2 | 9 | 0.08 | ko00998 |
|  | Synthesis and degradation of ketone bodies | 7 | 0.06 | ko00072 |
|  | Glycosphingolipid biosynthesis - ganglio series | 7 | 0.06 | ko00604 |
|  | Lipoic acid metabolism | 6 | 0.05 | ko00785 |
|  | Betalain biosynthesis | 5 | 0.04 | ko00965 |
|  | Glucosinolate biosynthesis | 5 | 0.04 | ko00966 |
|  | Polyketide sugar unit biosynthesis | 3 | 0.03 | ko00523 |
|  | Glycosphingolipid biosynthesis - lacto and neolacto series | 2 | 0.02 | ko00601 |
|  | Indole alkaloid biosynthesis | 2 | 0.02 | ko00901 |
| Organismal Systems | Plant-pathogen interaction | 397 | 3.40 | ko04626 |
|  | Circadian rhythm - plant | 131 | 1.12 | ko04712 |
